# Comparison of Corneal Biomechanical Properties between Post-LASIK Ectasia and Primary Keratoconus

**DOI:** 10.1155/2020/5291485

**Published:** 2020-10-26

**Authors:** Wuxiao Zhao, Yang Shen, Weijun Jian, Jianmin Shang, Vishal Jhanji, Aruma Aruma, Xingtao Zhou

**Affiliations:** ^1^Department of Ophthalmology and Optometry, Eye & ENT Hospital, Fudan University, Shanghai, China; ^2^NHC Key Laboratory of Myopia, Fudan University, Shanghai, China; ^3^Shanghai Research Center of Ophthalmology and Optometry, Shanghai, China; ^4^UPMC Eye Center, Department of Ophthalmology, University of Pittsburgh School of Medicine, Pittsburgh, PA, USA

## Abstract

**Purpose:**

To compare the corneal biomechanical properties between post-LASIK ectasia and primary keratoconus.

**Methods:**

A total of 42 eyes of 42 patients with matching age and central corneal thickness (CCT) were divided into two groups according to diagnosis of post-LASIK ectasia (PLE group; *n* = 21; age range: 22–47 years) and primary keratoconus (KC group; *n* = 21; age range: 21–47 years). The corneal biomechanical properties were assessed using Scheimpflug-based technology (Corvis ST; Oculus Optikgeräte, Wetzlar, Germany). The paired *t*-test and linear regression analysis were performed.

**Results:**

The PLE group had significantly higher mean stiffness parameter at the first applanation (SP-A1; 76.65 ± 21.66 vs 52.72 ± 13.65, *p* ≤ 0.001) and mean stress-strain index (SSI) (SSI: 0.78 ± 0.16 versus 0.64 ± 0.12, *p*=0.001) than the KC group. SP-A1 was positively correlated with CCT in the PLE group (Pearson's *r* = 0.816, *p* ≤ 0.001), but not in the KC group (Pearson's *r* = −0.014, *p*=0.952). No statistical correlation was observed between SSI and CCT in either group (Pearson's *r* = 0.292, *p*=0.199, and Pearson's *r* = 0.004, *p*=0.985, respectively).

**Conclusions:**

In our case series, KC manifested more severe than PLE in biomechanical properties. Since SSI measurements were independent of corneal thickness, it can be used for corneal biomechanical assessment.

## 1. Introduction

Post-LASIK ectasia refers to iatrogenic keratectasia following corneal refractive surgery, most commonly following laser in situ keratomileusis (LASIK). It is associated with abnormal preoperative corneal topography, excessive laser ablation, and altered corneal biomechanical properties [1]. Post-LASIK ectasia resembles primary keratoconus in certain aspects such as abnormal posterior elevation, corneal steepening, corneal thinning, and breaks in Bowman's membrane [2–4]. Corneal biomechanical decompensation is considered to be a key trigger for collapse of the structural stability especially in eyes with post-LASIK ectasia [5]. It has been reported that alteration in corneal biomechanical parameters precedes the changes in corneal topography [6–8].

Thus far, corneal topography and corneal biomechanical analysis are the mainstream methods for diagnosis of corneal ectasia. An anterior segment analyser (Pentacam HR; Oculus Optikgeräte, Wetzlar, Germany) combined with a corneal biomechanical analyser (Corvis ST; Oculus Optikgeräte) demonstrated high sensitivity and specificity for early detection of both post-LASIK ectasia and primary keratoconus [8–11]. Biomechanical weakness has been reported in post-LASIK ectasia and primary keratoconus [12, 13]. However, whether there is a difference in corneal biomechanics between post-LASIK ectasia and primary keratoconus is unknown. A large number of proteins and protein classes are involved in the development of keratoconus [14], and these pathological changes may lead to a change in corneal biomechanics. The current study aimed to investigate the differences in biomechanical properties between post-LASIK ectasia and primary keratoconus.

## 2. Materials and Methods

### 2.1. Study Design

In this retrospective study, case charts of patients were reviewed at the Eye and ENT Hospital of Fudan University from December 2014 to December 2019. A total of 42 eyes of 42 patients (21 primary keratoconus, 21 post-LASIK ectasia) with matching age and central corneal thickness (CCT) were included. Keratoconus was classified based on disease severity as KC1, KC2, and KC3 as per the Topographical Keratoconus Classification. The study followed the tenets of the Declaration of Helsinki and was approved by the ethics committee of the Eye and ENT Hospital (approval no. ky2012-017).

### 2.2. Inclusion and Exclusion Criteria

The diagnosis of post-LASIK ectasia and primary keratoconus was made according to the criteria set in the global consensus on keratoconus and ectatic disease [2]. The interval between LASIK surgery and diagnosis of ectasia was between 2 and 18 years. The criteria for patient matching between groups were age difference ≤2 years and CCT difference ≤10 *μ*m. Patients with any ocular problems except keratoconus and history of LASIK were excluded.

### 2.3. Ophthalmological Examination

All patients underwent ophthalmological examinations including slit lamp biomicroscopy, corneal topography (Pentacam HR; Oculus Optikgeräte), assessment of biomechanical properties (Corvis ST; Oculus Optikgeräte), manifest refraction, and fundus photography. All examinations were performed by an experienced technician, and only images with acceptable quality were recorded for analysis [9]. A research version of Corvis ST (Oculus Optikgeräte, Wetzlar, Germany) was used in the study. Corneal topographic parameters, including flat keratometry (K1), steep keratometry (K2), mean keratometry (Km), maximum keratometry (*K*_max_), astigmatism of corneal front surface (Ka), and corneal anterior elevation and posterior elevation were obtained from the Pentacam (Table 1).

**Figure 1:**
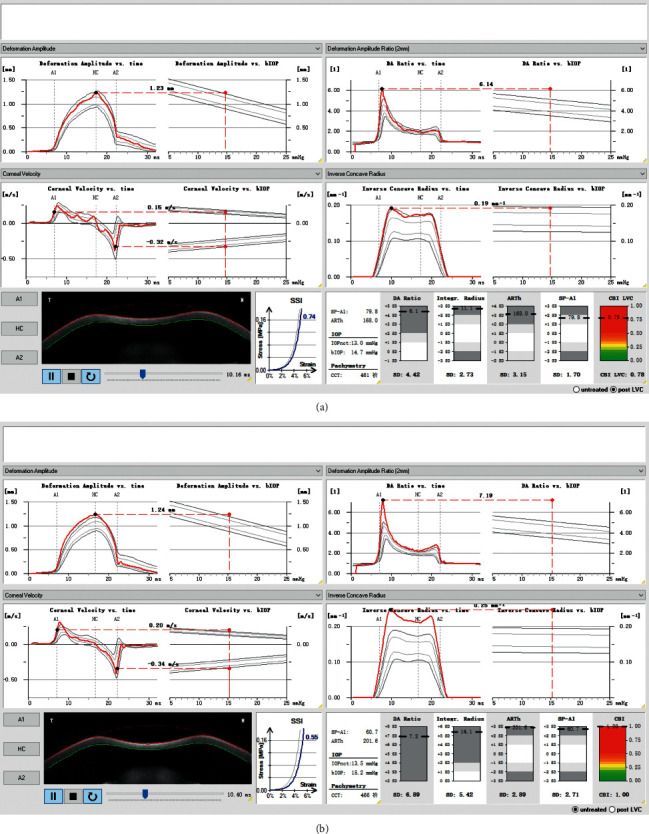
Vinciguerra Screening Report showing calculation of stiffness parameters (first applanation (SP-A1) and stress-strain index (SSI)) for post-LASIK ectasia (a) and primary keratoconus (b) with matching age and central corneal thickness.

**Figure 2:**
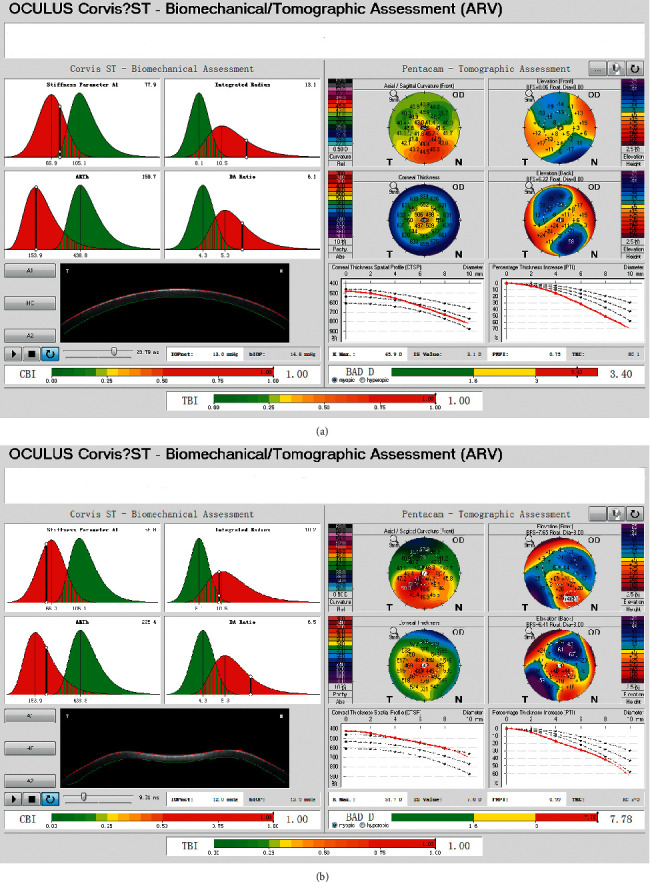
Biomechanical/topographic assessments (ARV) showing Corvis Biomechanical Index (CBI), Belin/Ambrósio Enhanced Ectasia Deviation Index (BAD-D), and Tomographic and Biomechanical Index (TBI) for the post-LASIK ectasia (a) and primary keratoconus (b) with matching age and central corneal thickness.

The following Corvis ST data were extracted: dynamic corneal response (DCR) parameters, including the first applanation length (A1 length), second applanation length (A2 length), velocity in (A1 velocity), velocity out (A2 velocity), peak distance, concave radius of curvature (radius), and deformation amplitude (DA); noncontact tonometer intraocular pressure (IOPnct), biomechanically corrected IOP (bIOP), deformation amplitude ratio (DA ratio), Ambrósio relational thickness to the horizontal profile (ARTh), stiffness parameter at the first applanation (SP-A1), and stress-strain index (SSI) (Figure 1); Corvis Biomechanical Index (CBI), Belin/Ambrósio Enhanced Ectasia Deviation (BAD-D) Index, and Tomographic and Biomechanical Index (TBI) (Figure 2).

### 2.4. Statistical Analysis

Statistical analysis was performed using SPSS Statistics for Windows version 23.0 (IBM Corp., Armonk, NY, USA). The Kolmogorov–Smirnov test was used to determine whether each parameter was normally distributed. Data included in this study were presented as mean ± standard deviation (normally distributed data). The paired *t*-test was used to compare the mean values of each parameter between post-LASIK ectasia and primary keratoconus groups. Pearson's correlation coefficient and linear regression analysis amongst the biomechanical parameters were conducted within the groups. Regarding multiple comparisons, Bonferroni correction was added, and *p* < 0.0029 was considered statistically significant.

## 3. Results

**Figure 3:**
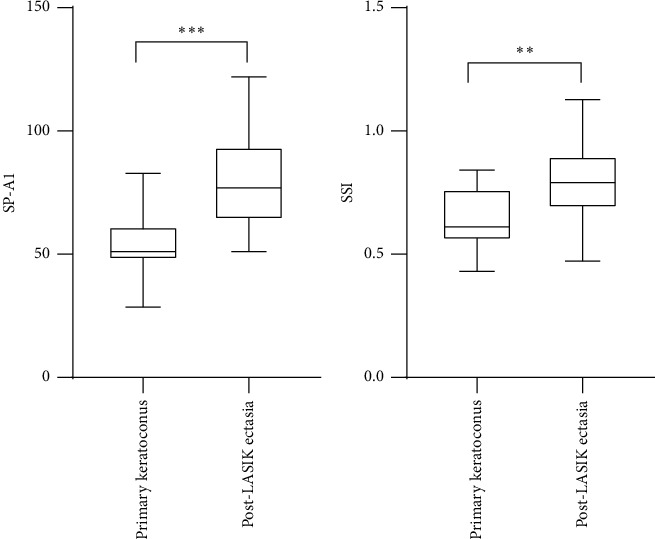
Significant differences in stiffness parameter at the first applanation (SP-A1, left) and the stress-strain index (SSI, right) between the post-LASIK ectasia and primary keratoconus groups (*p* ≤ 0.001, *p*=0.001, ^∗^^∗^^∗^*p* ≤ 0.001, and ^∗^^∗^*p* = 0.01  respectively).

The age- and CCT-matched patients with post-LASIK ectasia and primary keratoconus are shown in Table 2. The Kolmogorov–Smirnov test indicated that all Corvis ST parameters measured showed normal distribution. DCR parameters (A1 length, A2 length, A1 velocity, A2 velocity, peak distance, radius, and deformation amplitude), Vinciguerra Screening Report, and biomechanical/topographic parameters were calculated for post-LASIK ectasia (Table 3) and primary keratoconus group (Table 4). Significant differences were noted in SP-A1 (*p* < 0.001) and SSI (*p* < 0.01) between the PLE and KC groups (Figure 3).

**Figure 4:**
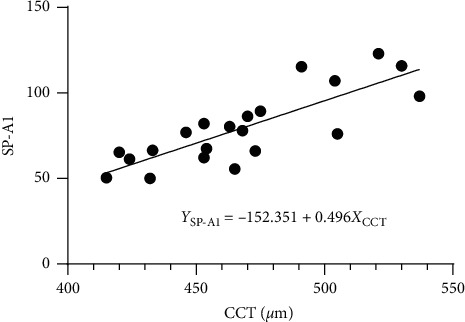
Stiffness parameter at the first applanation (SP-A1) as a function of central corneal thickness (CCT) in post-LASIK ectasia patients (linear regression, *F* = 37.760, *R*^2^ = 0.665, *p* ≤ 0.001).

A significant relationship between SP-A1 and CCT was noted in the PLE group (Pearson's *r* = 0.816, *p* ≤ 0.001), but not in the KC group (Pearson's *r* = −0.014, *p*=0.952). Moreover, no statistical correlation was observed between SSI and CCT in either group (PLE group: Pearson's *r* = 0.292, *p*=0.199; KC group: Pearson's *r* = 0.004, *p*=0.985). The linear regression function between SP-A1 and CCT was Y_SP-A1_ = −152.351 + 0.496X_CCT_ (*F* = 37.760, *R*^2^ = 0.665, *p* ≤ 0.001; Figure 4).

## 4. Discussion

The current study investigated the differences in biomechanical properties between post-LASIK ectasia and primary keratoconus using Scheimpflug imaging. SP-A1 was significantly lower in primary keratoconus than in post-LASIK ectasia, which suggested greater resistance to deformation in post-LASIK ectasia than in primary keratoconus. SP-A1 is defined as the resultant pressure (adjusted pressure at A1 minus bIOP) loading on the cornea divided by corneal deflection amplitude at A1 [9]. Previous studies demonstrated similar “interfiber fracture” in primary keratoconus and post-LASIK ectasia corneas [4]. However, in contrast to primary keratoconus, these histopathological and ultrastructural changes occurred only in the stress-bearing areas, corresponding to the stromal bed in post-LASIK corneas [15]. In primary keratoconus, changes in the expression of corneal epithelium and stroma-specific genes at the apex of the cone result in focal weakening in keratoconus [16]. Also, changes in keratoconus-related proteome occur in the noncone regions of keratoconus corneas [17].

In this study, we found a positive relationship between SP-A1 and CCT in post-LASIK ectasia, but not in primary keratoconus. It has been reported that the stiffness parameter is positively correlated with corneal pachymetry in both keratoconus and normal eyes, and the stiffness parameter values are significantly lower in keratoconus than in normal eyes with similar bIOP [18]. The absence of relationship between SP-A1 and CCT in primary keratoconus may be, at least partly, attributed to lower SP-A1 values (52.72 ± 13.65 and 76.65 ± 21.66 mmHg/mm for primary keratoconus and post-LASIK ectasia, respectively) in our study compared to the values (68.67 ± 23.64 and 108.10 ± 20.52 mmHg/mm for keratoconus and normal eyes, respectively) in a previous study [18]. Besides, patients with keratoconus had significantly higher keratometry values compared to post-LASIK ectasia, indicating a worse tomography and biomechanical decompensation in primary keratoconus.

We also noted that the SSI was significantly lower in the KC group than in the PLE group, indicating greater resistance to deformation in post-LASIK ectasia than in primary keratoconus. SSI is a newly described parameter, which can establish the whole stress-strain curve of corneal tissue and determine the corneal biomechanical properties in vivo [19]. The slope of the SSI is considered to reflect the tensile elastic modulus, with a higher slope representing a less strain-to-stress ratio and stiffer material. It has been shown that SSI is independent of bIOP or CCT but is significantly correlated with age in healthy eyes [19].

Our study has some limitations. In addition to a small sample size, patients in both groups were matched for age and CCT but not corneal topography. Furthermore, we did not have access to pre-LASIK clinical and topography data in the PLE group. In our case series, the KC group was more severe than the PLE group. We described the relationship between different parameters in both groups independently. Future studies can also focus on correlation between flap thickness and corneal stiffness parameters. Overall, the current study found that primary keratoconus was more severe than post-LASIK ectasia in term of corneal stiffness.

## Figures and Tables

**Table 1:** Demographic and topographic characteristics of the participants.

Characteristics	PLE group (*n* = 21)	KC group (*n* = 21)	*p*
Male/female ratio	17/4	14/7	0.484
Eye (right/left)	9/12	15/6	0.118
Age (years)	29.52 ± 6.00	29.24 ± 6.16	0.284
K1 (D)	41.55 ± 2.59	48.2 ± 5.3	≤0.001^*∗*^
K2 (D)	44.02 ± 4.41	51.9 ± 6.0	≤0.001^*∗*^
Km (D)	42.72 ± 3.15	49.7 ± 5.5	≤0.001^*∗*^
*K*_max_ (D)	48.47 ± 5.76	59.1 ± 8.8	≤0.001^*∗*^
Ka (D)	1.86 ± 1.58	4.18 ± 2.59	≤0.001^*∗*^
Anterior elevation (*μ*m)	12.48 ± 11.91	29.81 ± 13.33	≤0.001^*∗*^
Posterior elevation (*μ*m)	30.67 ± 23.63	61.43 ± 24.50	≤0.001^*∗*^
KC1, KC2, KC3	15, 4, 2	4, 7, 10	0.002^*∗*^
CDVA (logMAR)	0.114 ± 0.233	0.424 ± 0.391	≤0.001^*∗*^

PLE, post-LASIK ectasia group; KC, primary keratoconus group; K1, flat keratometry; K2, steep keratometry; Km, mean keratometry; *K*_max_, maximum keratometry; Ka, astigmatism of corneal front surface, KC, keratoconus severity according to Topographical Keratoconus Classification; CDVA, corrected distance visual acuity. ^*∗*^Significant difference.

**Table 2:** Age and central corneal thickness comparison between PLE and KC groups.

Parameters	PLE group	KC group	Paired differences	*t*	*p*
Age (years)	29.52 ± 6.00	29.24 ± 6.16	−0.286 ± 1.189	−1.101	0. 284
CCT (*μ*m)	468.19 ± 35.65	467.90 ± 33.89	−0.286 ± 3.594	−0.364	0.719

CCT, central corneal thickness based on Corvis ST; PLE, post-LASIK ectasia group; KC, primary keratoconus group.

**Table 3:** Corvis ST combined with Pentacam for corneal biomechanical analysis in post-LASIK ectasia.

Corneal biomechanical parameters	Mean	Standard deviation
*Dynamic corneal response*		
A 1 length (mm)	2.01	0.39
A 2 length (mm)	1.46	0.34
A1 velocity (m/s)	0.16	0.03
A 2 velocity (m/s)	−0.28	0.05
Peak distance (mm)	5.13	0.24
Radius (mm)	5.37	0.78
Deformation amplitude (mm)	1.14	0.11

*Vinciguerra screening report*		
IOPnct (mmHg)	13.02	1.75
bIOP (mmHg)	14.71	1.75
DA ratio	6.08	1.11
Integrated radius (mm)	12.30	2.27
ARTh	165.08	51.08
SP-A1	76.65	21.66
SSI	0.78	0.16

*Biomechanical/topographic assessment*		
Post-LVC CBI	0.99	0.02
BAD-D	5.49	3.63
TBI	0.87	0.24

A1 length, first applanation length; A2 length, second applanation length; A1 velocity, velocity in; A2 velocity, velocity out; radius, concave radius of curvature; IOPnct, noncontact tonometer IOP; bIOP, biomechanically corrected IOP; DA ratio, deformation amplitude ratio; ARTh, Ambrósio relational thickness to the horizontal profile; SP-A1, stiffness parameter at the first applanation; SSI, stress-strain index; post-LVC, post-laser vision correction; CBI, Corvis Biomechanical Index; BAD-D, Belin/Ambrósio Enhanced Ectasia Deviation Index; TBI, Tomographic and Biomechanical Index.

**Table 4:** Corvis ST combined with Pentacam for corneal biomechanical analysis in primary keratoconus.

Corneal biomechanical parameters	Mean	Standard deviation
*Dynamic corneal response*		
A1 length (mm)	1.81	0.30
A2 length (mm)	1.52	0.42
A1 velocity (m/s)	0.18	0.03
A2 velocity (m/s)	−0.31	0.05
Peak distance (mm)	5.07	0.26
Radius (mm)	4.97	0.73
Deformation amplitude (mm)	1.21	0.11

*Vinciguerra screening report*		
IOPnct (mmHg)	13.95	2.67
bIOP (mmHg)	15.63	2.91
DA ratio	5.94	0.97
Integrated radius (mm)	13.18	1.93
ARTh	187.14	69.68
SP-A1	52.72	13.65
SSI	0.64	0.12

*Biomechanical/topographic assessment*		
CBI	0.99	0.02
BAD-D	10.39	4.94
TBI	1.00	0.00

A1 length, first applanation length; A2 length, second applanation length; A1 velocity, velocity in; A2 velocity, velocity out; radius, concave radius of curvature; IOPnct, noncontact tonometer IOP; bIOP, biomechanically corrected IOP; DA ratio, deformation amplitude ratio; ARTh, Ambrósio relational thickness to the horizontal profile; SP-A1, stiffness parameter at the first applanation; SSI, stress-strain index; CBI, Corvis Biomechanical Index; BAD-D, Belin/Ambrósio Enhanced Ectasia Deviation Index; TBI, Tomographic and Biomechanical Index.

## Data Availability

Data analyzed in the current study are available from the corresponding author upon reasonable request.
